# Clinical implementation of the AAPM Task Group 36 recommendations on fetal dose from radiotherapy with photon beams: A head and neck irradiation case report

**DOI:** 10.1120/jacmp.v1i1.2650

**Published:** 2000-01-01

**Authors:** Karl L. Prado, Stephen J. Nelson, Joost J. Nuyttens, Todd E. Williams, Kenneth N. Vanek

**Affiliations:** ^1^ Radiation Oncology Department Medical University of South Carolina 171 Ashley Avenue Charleston South Carolina 29425

**Keywords:** fetal radiation dose, radiation therapy, pregnant patient radiation therapy

## Abstract

We present the results of our efforts in estimating and diminishing the fetal dose expected when a 29‐year‐old patient, 22 weeks pregnant, received external beam radiation therapy for a squamous cell carcinoma of the tongue. We explain our use of the information contained, and recommendations made, in the Report of the American Association of Physicists in Medicine Radiation Therapy Committee Task Group 36 [Med. Phys. 22, 63–82 (1995)]. We also explain our dose estimation, describe our validation measurements, and demonstrate the effectiveness of supplemental shielding. Consequently, this case report will serve as a guide to radiation oncologists and medical physicists who may encounter similar cases.

PACS number(s): 87.53.–j, 87.52.–g

## INTRODUCTION

Provision of radiation therapy to a pregnant patient for whom treatment is indicated presents several challenges. Treatment must be delivered accurately and in a timely fashion while maximum protection against radiation is afforded to the embryo‐fetus. Preparation for treatment involves estimating the expected fetal dose, devising appropriate shielding, assessing its effectiveness, and then measuring the resultant fetal dose. Ideally, this dose is maintained below levels at which deleterious effects may appear. Task Group 36 of the Radiation Therapy Committee of the American Association of Physicists in Medicine (AAPM TG‐36) has published, in its report “Fetal Dose from Radiotherapy with Photon Beams,”^1^ clear guidance for this process. We describe herein our implementation of the recommendations of that report in the case of a patient, 22 weeks pregnant, who received head and neck radiation therapy.

Our patient was diagnosed as having a pT2 pN2b M0 left mobile tongue squamous cell carcinoma. She underwent a left perioral glossectomy with left supraomohyoid neck dissection when 16 weeks pregnant. Radiotherapy was begun six weeks later. The upper neck was treated to 50 Gy with two lateral 6‐MV *x*‐ray fields; spinal cord blocking was introduced at 44 Gy and bilateral 9‐MeV electron posterior neck fields were begun. After 50 Gy, the lateral fields were reduced and the tongue was boosted to 60 Gy. The left neck was treated with 9‐MeV electrons to a total dose of 64 Gy. The lower neck was treated to 50 Gy with a single anterior 6‐MV *x*‐ray supraclavicular field. The left supraclavicular region was then boosted to 64 Gy with 9‐MeV electrons.

In the study reported here, we calculated the expected total fetal dose and validated the estimate through phantom and patient measurements. We also devised supplemental fetal shielding and evaluated its effectiveness. Only fetal doses from photon fields were considered in this case report since the additional dose to the fetus contributed by the electron fields was essentially negligible. On the basis of data published by Antolak and Strom^2^ pertaining to fetal doses resulting from chest‐wall electron fields, the additional fetal dose that could be expected from the electron fields used in this case is estimated to be no more than 0.1–0.2 cGy.

## METHODS

### Estimation and measurement of fetal dose

The arrangement of treatment portals was selected prior to patient simulation. The initial fields were to consist of two lateral upper‐neck fields and an anterior lower‐neck field. We chose to use our standard “single‐isocenter” technique.^3^ We also elected to estimate fetal dose at two points, representing the maximum and minimum expected. The first (maximum) point was located at the uterine fundus, 41‐cm inferior to the point chosen for the placement of the treatment fields' common isocenter; the second (minimum) point was located at the pubis, 55‐cm inferior to the isocenter.

The doses expected at each of these points from each of the treatment fields were calculated using peripheral dose (PD) data contained in the AAPM TG‐36 report.^1^ Peripheral dose (or “off‐axis dose,” the term used in the TG‐36 Report) describes the radiation dose at a certain distance outside of a treatment field normalized to the dose existing at the depth $\left(d_{\max}\right)$ of maximum dose on the central axis of the treatment field. The PD at a point of interest varies with radiation energy, distance to the closest edge of the treatment field, field size, and, to a much lesser degree, depth.^1^ ^,^ ^4^ ^,^ ^5^ The TG‐36 report presents PD data that summarize measurements made by various investigators. PD data are presented in graphical form as a function of distance to the field edge.

Since, in the AAPM TG‐36 report, PD is expressed as a percentage of the dose at $d_{\max}$ along the central axis of the treatment field, it was necessary to calculate the dose at this point from knowledge of the dose at the field's prescription point. Appropriate inverse‐square, tissue maximum ratio (TMR), and off‐axis corrections were applied to each field's prescribed dose to obtain the field's $d_{\max}$ dose. The dose expected from each field was calculated individually; these doses were then summed to produce a dose equivalent to that generated by the treatment, in a single fraction, of the combined left and right lateral and anterior supraclavicular fields.

Calculated doses were verified by measurement. Thermoluminescent dosimeters (TLDs) made of lithium fluoride were used to measure the dose at the fetal‐dose estimate points. The TLDs were placed on a RANDO anthropomorphic phantom (Alderson Research Laboratories, Stamford, CT) at distances from the treatment fields corresponding to the maximum and minimum fetal‐dose points. Measurements were made on a Varian Clinac 2100C accelerator (Varian Medical Systems, Des Plaines, IL). At each measurement distance, two sets of TLDs were used: (1) the first was placed at a depth of 10 cm in the phantom; (2) the second was placed on the surface of the phantom under a 1.0‐cm slab of flexible tissue‐equivalent bolus. TLD measurements at depth are representative of fetal dose, while those at the surface relate the dose at depth to that measured at a point at which patient dose can be monitored. The phantom was irradiated, without supplemental shielding, using the treatment geometry utilized for our patient. Monitor‐unit settings equal to five days of treatment were used. The process was then repeated using fetal shielding to evaluate its effectiveness. Finally, patient dose was measured by placing TLDs on the patient's skin, under 1 cm of bolus material, at the points of expected maximum and minimum fetal dose.

### Design of supplemental shielding

Supplemental shielding should afford maximal fetal radiation protection consistent with patient safety and ease of placement. In addition, the design of an effective shield should take into consideration the source of radiation. As the distance between the dose‐estimate point and the treatment fields increases, the relative contribution of head leakage to the total dose increases. Data by Kase and others^4^ seem to indicate that for 6‐MV radiation, leakage contributes approximately half of the dose to a point 40 cm from the edge of a $10\times 10\;{\text{cm}}^{2}$ treatment field. Scatter from the collimators and from the irradiated volume itself becomes more significant as the dose‐estimate point approaches the treatment field. In head and neck irradiation situations, where treatment‐field distances to the fetus are 30–50 cm, shielding against leakage becomes a primary goal. This simplifies the shielding problem since one can assume the source of leakage is the *x*‐ray target and design shielding to protect the fetus from radiation originating at that point. Shields devised for collimator scatter will have, by necessity, a greater cross‐sectional area, and hence greater weight, since scatter originates in the entire collimator assembly including field‐shaping blocks.

**Figure 1:**
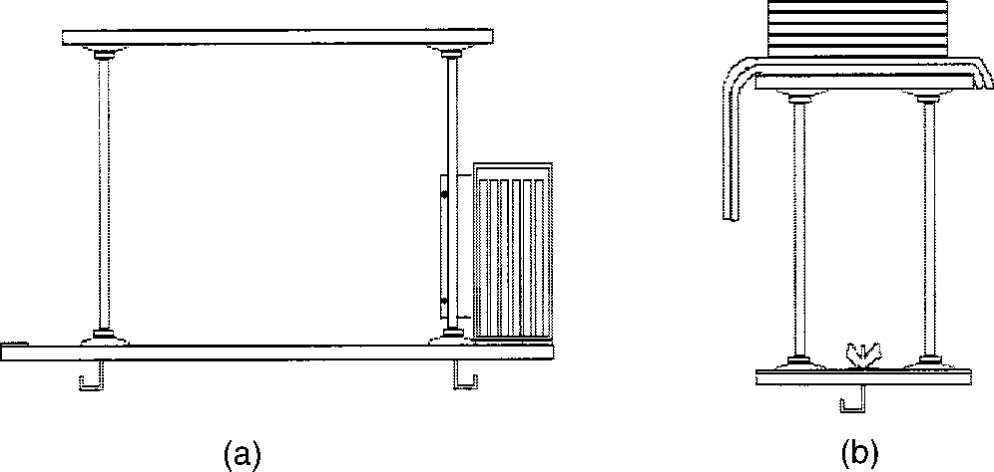
(a) Supplemental shielding device as viewed from the foot of the treatment table as one looks at the gantry. Head‐leakage shields are shown in position for the treatment of a left lateral field. (Patient is supine; for clarity, patient, scatter aprons, and couch are not shown.) (b) Supplemental shielding device as viewed from the (patient's right) side of the treatment table. The shielding configuration shown is that used for the treatment of the anterior field (the gantry would be located on the top left of the figure). Scatter apron and leakage shielding are shown.

Our shielding device (Fig. 1) was designed with the aforementioned considerations in mind. It is comprised of shielding materials and a shielding holder. The shielding materials consist of two parts. Head‐leakage shielding consists of low‐melting‐point alloy plates 25‐cm square and 1.5‐cm thick cast within electron molds. This casting yielded plates weighing 10 kg, which could be easily stacked and handled by the therapists. A total of six plates was deemed satisfactory to attenuate head leakage. The scatter shield consists of lead sheet aprons cut from a 1/16th‐inch‐thick lead roll 4‐ft square. Each sheet was cut into four equal parts and then layered on top of each other and contoured to the shielding holder. The edges of the drape were taped together so that the sheets become one unit weighing approximately 21 kg. Two overlapping aprons were fabricated in this fashion and were painted with spray paint to minimize contact exposure to the lead.

The shielding holder is an adaptation to the accelerator treatment table. The basic device consists of two pieces of $\frac{3}{4}$‐inch birch plywood whose dimensions allow the device to reach across the “H” bars of the accelerator couch and support the necessary shielding materials. The upper piece is supported by four galvanized steel pipes and eight mounting flanges screwed to the plywood. Two screw hooks that clasp onto the accessory rails of the couch provide stability. The plywood is essentially the same thickness of the panels on the treatment couch, so there is no discomfort to the patient as she is positioned.

For the lateral fields, the low‐melting‐point alloy plates are stacked on edge in a frame constructed from aluminum angle and flat stock. This frame holds the plates by resting on the plywood just off the side of the couch resembling a “saddle bag.” Horizontal stability is provided by thumbscrews that lock the frame to the two pipes on that side. The frame is symmetrical front to back such that it can be used on either side of the couch. Consequently, the aluminum saddlebag is first placed onto the beam side of the shield support table, locked into place, and then loaded with the plates. The plates are positioned such that they cast a “protective shadow” originating from the target of the accelerator and covering the patient's fetal area.

## RESULTS

The peripheral doses existing at our dose‐estimate points from the anterior and lateral fields were obtained from Figs. 19 and 21 of the TG‐36 report. The dose per fraction at each point from each field was calculated from its corresponding PD utilizing the field's $d_{\max}$ dose per fraction. The results of these calculations are shown in Table I where, for each measurement point, distances to and PDs from each treatment field are shown. Also shown are expected unshielded doses at each point per fraction. Note that the dose shown at each point from a lateral field represents the dose existing at the point from the treatment of a single lateral field. The dose per fraction at each point from the combined pair will equal twice the dose of the single lateral.

**Table I:** Dose‐estimate points, distances to treatment fields, peripheral doses at each point from each field, and dose per fraction at each point from each field.

	Dose‐estimate point			
	Maximum		Minimum	
Treatment field	Anterior	Lateral^a^	Anterior	Lateral^a^
Distance to field edge (cm)	31	41	45	55
Peripheral dose (%)	0.112	0.060	0.043	0.037
Dose per fraction (cGy)	0.232	0.067	0.089	0.041

a The dose per fraction from the lateral field is the dose per single lateral field. The dose from the combined pair of lateral fields equals twice the dose from the single field.

Table II shows the expected total unshielded doses per fraction at each point. Our calculations are compared to our 10‐cm‐depth TLD measurements for the unshielded phantom. In this table, dose per fraction represents the dose resulting from the treatment of all three fields (anterior and two laterals). Note that although the percent differences may appear to be large, the magnitude of the differences is maximally about 0.1 cGy.

**Table II:** Calculated maximum and minimum expected unshielded fetal doses per treatment fraction and their comparison with measured unshielded fetal doses. (One standard deviation is shown for the measured data.) One treatment “fraction” consists of three fields: right and left lateral fields plus the anterior supraclavicular field.

Dose‐estimate point	Total calculated expected dose (cGy)	Total measured dose (cGy)	Percent difference^a^
Maximum	0.37	0.47±0.03	27.0
Minimum	17.6	0.20±0.04	17.6

a Percent difference=100×(measured‐expected)/expected.

Shielding effectiveness is shown in Table III. Dose reduction was determined by obtaining the difference between the 10‐cm‐depth TLD measurement at each dose‐estimation point with and without shields in place. Percent reductions were then obtained by dividing the differences by their corresponding unshielded values. It should be noted that the anterior “apron shield” was left in place for all fields while the “leakage shields” were moved between fields.

**Table III:** Effectiveness of supplemental shielding at the maximum and minimum dose estimate points. One treatment “fraction” consists of three fields: right and left lateral fields plus the anterior supraclavicular field.

Dose‐estimate point	Dose/fraction without shields (cGy)	Dose/fraction with shields (cGy)	Dose reduction (%)
Maximum	0.474	0.273	42.4
Minimum	0.202	0.079	60.9

The results of patient measurements are shown in Table IV, where doses for the entire course of treatment have been totaled. The fractional dose contributed by the lateral boost fields to each dose‐estimation point was determined from Table I and then multiplied by the number of boost fractions. The resultant dose was then added to the dose from the combined lateral‐anterior field combination to obtain a total for the entire course of treatment. Note that this overestimates the fetal dose slightly since the data of Table I do not account for the dose reduction produced by the supplemental shielding. In Table IV, phantom measurements consist of the results of the TLDs placed at depth in the RANDO phantom. Patient measurements incorporate the results of the TLDs placed under a bolus on the patient's skin; dose at depth was obtained by multiplying the skin measurement by the ratio of depth‐to‐skin TLD results obtained in our phantom study.

**Table IV:** Maximum and minimum total estimated fetal doses after treatment of all photon fields. Estimates are differentiated on the basis of both patient and phantom measurements.

Dose‐estimate point	Total dose predicted by patient measurements (Gy)	Total dose predicted by phantom measurements (Gy)
Maximum	0.085	0.090
Minimum	0.054	0.027

## DISCUSSION

The AAPM TG‐36 report proved to be an excellent resource in this fetal‐dose estimation endeavor. The guidance was very appropriate and the data were extremely useful. The agreement between calculated and measured estimated doses as shown in Table II is quite good particularly when one considers all the possible sources of uncertainty that may exist. In fact, while there are numerous assumptions underlying the PD data of the TG‐36 report that may not be specifically applicable in a particular clinical situation, it is comforting to discover that preliminary calculations yield reasonably accurate fetal‐dose estimates. The good agreement between calculation and measurement, on the other hand, does not preclude the need for measurements, particularly since the effect of supplemental shielding, which will vary in any clinical situation, cannot be easily predicted by calculation.

Shielding was designed and constructed to afford as much fetal radiation protection as possible while maintaining patient and staff safety, simplicity of fabrication, and ease of setup. The data of Kase *et al.* ^4^ which show the relative contribution of head leakage, patient scatter, and collimator scatter to the total PD, was most helpful. For this case, the shield was designed to attenuate primarily leakage radiation from the lateral fields and leakage plus collimator scatter from the anterior field. The shield effectively reduced the dose to the unshielded fetus by roughly 50%. The effectiveness of our shield, shown in Table III, appears to be consistent with the data of Kase *et al.* as well as with the fetal‐dose estimate examples shown in the TG‐36 report. Leakage begins to become the major source of PD at the distances between the fetus and the radiation fields that exist during head and neck irradiation. As seen in Table III, a 60.9% reduction of the unshielded dose was achieved at our minimum dose‐estimate point, while at the maximum dose‐estimate point, a reduction of 42.4% was attained. These data seem to suggest that in‐patient scatter and scatter from the collimator and field‐defining blocks are the main constituents of the dose at the maximum point, which is only 31 cm away from the anterior field. Thus, shielding designed to primarily attenuate leakage becomes less effective at that point.

The total estimated fetal dose appears to vary from 0.03 to 0.09 Gy, the variability being largely due to distance from the treatment fields. We cannot explain why such a relatively large difference exists between the doses predicted by patient and phantom measurements at the minimum point while excellent agreement exists between the two measurements at the maximum point. This difference, however, does not diminish the validity of the dose estimate since lower limits of dose estimates are rarely used.

Our maximum dose estimate is consistent with measurements made and reported by Sneed and others.^6^ In that report, the authors present the results of measurements made when a patient received lateral‐field head and neck radiotherapy during pregnancy. Fetal dose, in that report, was estimated to be approximately 0.09% of the dose at isocenter. Our maximum fetal dose represents 0.08% of the isocenter dose, where, for the purpose of our estimate, a dose of 60 Gy from the lateral fields plus 50 Gy from the anterior field exists. It is interesting to note that although fairly significant differences existed in the treatment conditions of their patient and ours (the authors' patient was treated with lateral fields only and no abdominal shielding, while our patient was treated with both lateral and anterior fields with additional abdominal shielding) reasonable agreement exists in our measurements. It is possible that the effects of the differences in treatment conditions “cancel out,” i.e., the use of additional shielding offsets the additional peripheral dose resulting from the closer anterior supraclavicular field.

The effects of radiation on the developing embryo‐fetus depend upon gestational age as well as on dose. During the initial stages of development, i.e., the preimplantation and embryonic stages, radiation effects are principally lethality and malformation of specific organs. During the intermediate early‐fetal and mid‐fetal stages, mental retardation and small head size become the main effects. After the onset of the late‐fetal stage, the risks of malformations and mental retardation become almost negligible. At this point, subsequent cancer development becomes the major risk.^1^

Doses lower than 0.1 Gy do not appear to produce an observable effect on fetal development as assessed by growth retardation, malformations, and mental deficiencies. At such low doses, the absolute risk of these effects to the fetus is minimal and would be almost impossible to separate from the underlying spontaneous congenital abnormality rate.^7^ ^,^ ^8^ Childhood cancer incidence appears to have an associated absolute risk of 6% per Gy.^9^ Assuming a maximum fetal dose of 0.09 Gy and fetal irradiation during the mid‐to‐late fetal stages, negligible risks exist for growth retardation, malformations, mental deficiencies, and induction of childhood malignancies.

## CONCLUSION

The pregnant patient can and should receive radiation therapy in a safe and effective manner. However, effort must be devoted to both estimating and diminishing fetal dose. This report has been written as a guide to radiation oncologists and medical physicists who may be faced with this situation. Although the case reported here applies specifically to radiation therapy of the head and neck area, the process for other anatomical areas is the same. The guidance and data of the AAPM TG‐36 report are most valuable in this regard. The process of accurately estimating fetal dose via calculations and measurements helps with shielding design and development. Shields can be constructed fairly easily, effectively, and inexpensively. Through their use, fetal doses can be reduced appreciably. In the case of head and neck irradiation, the use of simple shields can reduce the dose to relatively safe levels.
